# The Effects of the COVID-19 Pandemic on the Mass Market Retailing of Wine in Italy

**DOI:** 10.3390/foods10112674

**Published:** 2021-11-03

**Authors:** Francesca Gerini, Andrea Dominici, Leonardo Casini

**Affiliations:** Department of Agriculture, Food, Environment and Forestry, University of Florence, P.le delle Cascine 18, 50144 Firenze, Italy; francesca.gerini@unifi.it (F.G.); leonardo.casini@unifi.it (L.C.)

**Keywords:** coronavirus, scanner data, wine consumer, lockdown, supermarkets

## Abstract

The purpose of this study was to provide a detailed framework of wine purchases in supermarkets during the COVID-19 pandemic. The unexpected diffusion of the virus and the restrictions imposed in Italy to prevent its spread have significantly affected the food purchasing habits of consumers. By analyzing the scanner data of the wine sales in the Italian mass market retail channel, this study was intended to show whether and how the dynamics triggered by the pandemic have modified the overall value and type of wine purchases, focusing on prices, formats, and promotional sales. In particular, this study explores sales in two separate periods, namely March–April (the “lockdown”, with general compulsory closing and severe restrictions) and June–July 2020 (the “post-lockdown”, in which some limitations were no longer effective). The analysis of wine sales during lockdown and post-lockdown and the study of the variations compared to the sales of the previous years showed some significant changes in purchase behavior. The results could provide managers, researchers, and policy makers with extensive insights into the purchasing patterns of consumers during this unprecedented time and reveal trends that may characterize the structure of the future wine demand.

## 1. Introduction

In December 2019, various cases of pneumonia began to crop up in China [1]. Later identified as the new SARS-CoV-2 coronavirus (also called COVID-19), the disease rapidly spread all over the world, affecting more than 90 million people, and causing almost two million deaths in a year [2]. In Italy, after verifying the first two cases of COVID-19 on 30 January 2020, various restrictive measures were adopted, some of which were very rigid, in view of fighting the pandemic. Lockdowns, closing restaurants and bars, travel restrictions, and working from home were just a part of these limitations that have significantly affected food habits, the purchasing channels of food products, and even the contents of the shopping cart [3,4,5]. For example, in the period between 17 February and 3 May 2020, the value of sales in mass market retailing (MMR) observed by Nielsen, recorded a growth of +4.2% compared to the same period from the previous year [5].

The unexpected pandemic caused an unprecedented global crisis and disrupted food systems, changing regular food choices and habits, and making food even more central in customers’ lives [6,7,8,9]. The impact of the pandemic may have influenced wine purchase behavior, shifting the sales channel and changing the typology and characteristics of the wine that was bought, and therefore misaligning sales from the trends forecasted by previous studies. A study by Wittwer and Anderson [10] projected for 2020 a reduction of 14% in the value of the domestic consumption of wine in Western Europe due to COVID-19. In particular, within wine groups, they predicted −8% for wine with a price less than 2.5 USD/liter, −11% for wine in the range of 2.5–7.5 USD/liter, and −7% for wine priced over 7.5 USD/liter. The first report conducted in Italy by IRI [11] pointed out an increase in wine sales in the first ten months of 2020, both in the MMR channel (+6.9% in value), and especially in small grocery stores (+200%). At the same time, online wine sales also rose considerably. Even though, for Italians, the food e-commerce sector has always been the least popular compared to other goods and products [12], online wine sales recorded a +149% increase in value compared to the same period of 2019 [13]. The driving channels of this growth were the pure players (e.g., Tannico or Callmewine), which accounted for 83% of sales, and the e-shops of the MMR channel, with Amazon representing 17% of online wine sales [13].

This situation elicited the need to explore in depth the wine sales observed during the pandemic in the MMR channel. Did they increase compared to the past? In which price range did consumers purchase the most wine? What was the most purchased format during the pandemic? Have the wine promotion sales grown? Was there an increase in the prices of wine bottles? The purpose of this study was to answer these questions, providing a detailed framework for the different formats and price ranges chosen by customers during two different periods of the COVID-19 pandemic. Since the pandemic began, an initial period of compulsory closing called a “lockdown” (March–May 2020) was followed by a period in which limitations were no longer effective, and in Italy people returned to a general resumption of economic activities and free circulation among regions. Our analysis thus investigated the wine sales in these two different periods, namely, March–April (lockdown) and June–July 2020 (post-lockdown). The analysis applied in these two different timeframes provided several conclusions. Firstly, we compared the trend of total wine sales in March–April 2020 with the sales of the same two months in previous years. This examination allowed us to verify whether the unexpected lockdown caused a change in the consumer wine purchasing patterns. Moreover, the analysis was expanded to examine the data for the June–July 2020 period. The results of this period enabled us to explore the evolutions in wine purchases, namely, whether the sales trends taken on during the lockdown were maintained afterwards, and to observe if these trends showed variations compared to the same two-month period for previous years.

Although several previous studies have explored the changing trends in food purchases during the pandemic, analyzing the variations of sales compared to 2019 [14,15,16,17], there is a lack of information regarding wine purchases. In addition to representing a traditional element in food expenditures of Italians, wine is a suitable product to identify market trends in a period of crisis. In fact, not being an essential good, wine is characterized by a demand that is more sensitive to the socio-economic dynamics [18], such as that underway during this unprecedented pandemic. The sales of wine in Italy are traditionally pulled by the MMR, which represents the main distribution channel. In fact, according to a survey conducted by Mediobanca [19], in 2019, 36% of national sales of the principal wine-growing companies were made on the MMR channel. The second distribution channel was that of wholesalers/brokers (19.6%), followed by the hotel, restaurant, and catering (HoReCa) sector with 16.8%. Wine sales in wine shops and wine bars totalled 6.9%, whereas direct sales accounted for 11.6%. Direct sales also included online trading, which represented 1.1% on average. The rest of the wine sales were included in the category of “other” distribution channels. Despite the relevant role of MMR in the wine market, no prior studies have provided detailed insights into wine consumers’ purchases by considering the wine sales in this channel during the pandemic. Using store scanner data, this study explored whether the market for wine in supermarkets changed in two particular periods of the pandemic, characterized by different restrictions. Furthermore, the analysis of real wine sales data allowed us to verify whether the results reported by previous empirical studies are confirmed by real sales data.

The analysis of wine sales in MMR during the pandemic contributes to the literature by shedding light on how consumers change their purchasing patterns during an emergency crisis, which is unique in several aspects. In fact, though the crisis caused by COVID-19 may share some similarities with previous economic and financial crises, no previous pandemic has affected so many countries in such a short period of time or has caused a sudden global stop in economic activity induced by containment measures [20,21]. In this sense, the purchase behavior acquired during this period could have also long-term effects that may characterize the structure of the final demand for wine on the MMR channel even after the pandemic.

## 2. Study Overview and Research Questions

After the first two cases of COVID-19 on 30 January 2020, the Italian government started to take strong containment measures to prevent the spread of the pandemic. Following the first closings, localized in several areas of the country by the end of the month of February, a DPCM (Decree of the Prime Minister’s Office), dated 9 March, established several restrictions over the entire national territory. In addition to prohibiting travel (except for when motivated by self-certified reasons of work, situations of necessity, or health reasons) and all forms of assembly of people in public places, the decree also provided for the closing of schools, museums, theatres, and movie theatres, as well as prohibiting civil and religious ceremonies. Furthermore, in order to reduce the chance of contact between people, the DPCM provided for the closing of bars and restaurants at 6 pm, thereby preventing the possibility of on-premises dining. These measures were further reinforced by a new DPCM that entered into effect on 12 March, which suspended all retail sales activities, except for stores dealing in foodstuffs, basic requirements, and pharmacies. These restrictive measures were then followed by the closing of all public food-service establishments, including bars, restaurants, pubs, pizzerias, and catering. The DPCM, however, allowed the possibility for food service providers to make home deliveries. These adopted measures remained valid for about two months, that is, until the second half of the month of May.

**Figure 1:**
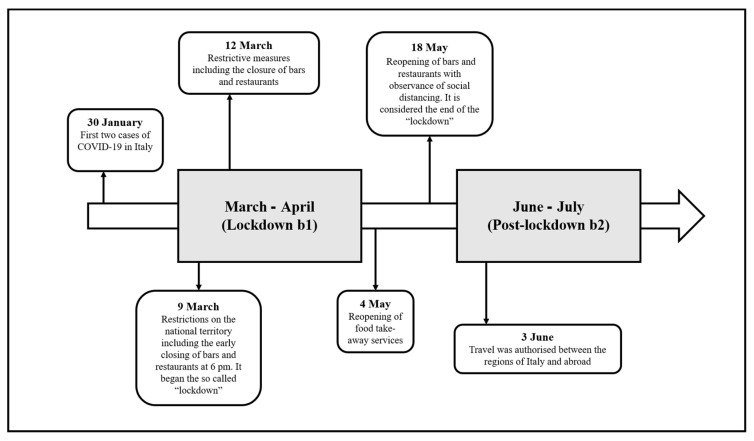
Timeline with the events occurring in Italy during the COVID-19 pandemic relevant for this study in 2020.

The first let-ups on restrictions were permitted by the DPCM that entered effect as of 4 May, which authorized food services on a take-away basis, prohibiting the consumption of products on the premises and parking in the immediate vicinity. The resumption of food service activities was permitted as of 18 May, limiting admission to a small number of customers, and adopting social distancing amongst them. As of the 3 June, travel was authorized between the regions of Italy and abroad. Figure 1 shows these data on a timeline.

The spread of the pandemic and the consequent restrictive measures adopted could have modified the market sales of wine. In Italy, MMR represents the first distribution channel for wine in terms of value and volume. Table 1 reports an overall picture of the Italian wine market for this distribution channel.

In general, the data in Table 1 show a general increase in the whole wine sales in MMR for 2020. Considering also that the sale of foodstuffs via MMR increased in the lockdown period [5], we intend to explore whether and how this change in purchase patterns also affected wine sales in the considered period. Hence, the first underlying research questions of this study are:

**Q1:**  *During the lockdown, how much were the wine sales in terms of value and volume on the MMR channel? Did these wine sales change compared to the past?*

For one segment of the population, the pandemic brought some economic difficulties. Compared to the same periods in 2019, the authorized unemployment benefits paid out were 16 times higher in March–April 2020 and 12 times higher in June–July 2020 [23]. In the second trimester of 2020, Italy recorded a decline in the number of employed people (−3.6%) compared to the same period of 2019 [24]. Economic difficulties may therefore have influenced food habits, especially regarding an increase in the sales of low-cost products. In a study on the changes in the food consumption patterns of Greek households due to the economic crisis, Duquenne and Vlontzos [25] showed that consumers preferred less expensive products, characterized by low prices. The *non-premium wines* or *basic wines*, which is to say wines with an average price of less than 3 EUR/liter [26], represented a preferred sales channel in the modern distribution [27]. According to Casini et al. [28], in 2017 *basic wine* represented 33.0% of wine sales in value. Although the consumption of *basic wine* was correlated with lower income consumers [27], the economic difficulties caused by the pandemic could have caused a decline in the purchasing power of families and a consequent increase in the sales of these wines. Furthermore, the period of lockdown saw an increase in home consumption, owing to the closing of most economic activities, in addition to the introduction of remote working [7]. This shift in consumption could have given rise to an increase in purchases of wine for daily consumption on the MMR channel and, therefore, of *basic wine*.

**Q2:**  *During the lockdown, in which price range did consumers purchase the most wine? What price ranges showed noticeable changes compared to the previous years?*

The range of *basic wines* is complex and characterized by marked heterogeneity [27]. In fact, the *basic wine* category includes wines in bottles or other packaging, such as plastic, carton (in Italy known as brik), bag-in-box, and demijohn packaging. Some of these formats may have recorded substantial increases in sales during this period for several reasons. On one hand, the limitations of movement imposed during the lockdown and the period immediately following it forced people to purchase large amounts of wine at neighbourhood supermarkets, such as the bag-in-box or demijohn, which previously were traditionally purchased directly from the winegrower or from wine retailers who were no longer accessible due to restrictions. Moreover, in order to avoid long queues to enter supermarkets and limit to possible opportunities of infection, the general trend of consumers was to reduce the number of times they went to the supermarket, preferring the purchase of foods and beverages in large amounts and with long best-before selling dates [29]. In this sense, the bag-in-box is a convenient packaging alternative. In addition to containing a large quantity and lasting longer, it maintains the wine’s sensory qualities longer than a glass bottle once it has been opened. Moreover, at the same volume, the bag-in-box packaging format or plastic prove to be more practical and easier to transport compared to glass bottles, which are at risk of breaking [30]. Therefore, our research questions are the following:

**Q3:**  *In the basic wine category, which were the most purchased wine formats? Which format showed the best performance compared to the past? Which format the worst?*

In Italian MMR, most of the sales in value are made in the 0.75 liter bottle format. In 2017, bottles sales accounted for EUR 1205.1 million and represented 75% of the total sales in value [28]. However, in MMR we can find bottles of wine with different prices and of different types. In fact, we can find bottles of *basic wine* priced under EUR 3/liter, and *premium* bottles priced over EUR 3/liter. The latter category is vast and includes even very expensive wines. We can imagine that the sale of wines belonging to different price ranges showed different trends during the lockdown. For example, the economic difficulties that emerged for several consumers and the trend towards saving could have driven the purchases of some consumers towards bottles in the *basic* range. At the same time, closing restaurants and wine bars prevented the customers of these establishments from consuming high-quality wine, in a higher price range, and often marked with indications of origin. Although in 2019 the HoReCa sector represented less than one-fifth of the overall wine market, wines that sold at more than EUR 25 reached 33.5% of the total sales [19]. This type of product that was previously consumed in restaurants could have entered homes more often during the lockdown. Moreover, the impossibility of celebrating special occasions with friends and relatives could have led to a reduction in the sales of the expensive wines [31,32,33]. For these occasions, the choice of wine often involved a bottle that also served to impress others [34]. In this sense, one could opt for a *great wine*, one that cost more than EUR 25 [35].

The pandemic could have influenced not only bottle prices, but also promotion policies. For food in general, Altroconsumo [36] has registered a general negative trend of promotional sales in Italian MMR. The large turnout of customers in supermarkets during the lockdown may have diminished the necessity of attracting consumers with promotional activities. At the same time, some supermarket chains could have decided not to change their promotional policies for ethical reasons.

Finally, the assortments of bottles available on the shelves of MMRs may have changed. Several wine producers, due to the closing of HoReCa and difficulties in exportation, entered new sales channels such as supermarkets [16,37,38]. Meanwhile, provision difficulties occurring during lockdown may have given rise to problems of widespread availability. These issues could have involved a change in the number of single items purchased by consumers. This number can be obtained by means of the EAN (European Article Number) code, that is, the barcode connected to every single item.

To explore the overall sales of wine bottles, our research question was the following:

**Q4:**  *During lockdown, what was the performance of the 0.75 liter glass bottles, exploring the total sales value, prices, promotion sales, and the number of EAN codes purchased?*

All the above research questions concern the purchasing behavior of wine consumers in the March–April 2020 period and its evolution compared to the same period in previous years. However, with the progressive reopening of commercial activities in mid-May, consumers may have changed their behavior again. In this sense, wine purchases made in June and July 2020 may have followed the trends observed during the lockdown or may have confirmed the trends of the years prior to the pandemic. We therefore asked:

**Q5:**  *In the post-lockdown period, which trends in the sales of various types of wine were in line with those of the lockdown? Which sales followed the evolution trend of previous years?*

## 3. Materials and Methods

In order to explore wine sales in depth and answer the proposed research questions, we used the IRI Infoscan database, which reports the scanner data of the sales of still and semi-sparkling wines (wines with an excess pressure, due to carbon dioxide in solution, of not less than 3 bar when kept at a temperature of 20 °C in closed containers) throughout Italy for the MMR channel, including superstores (hypermarkets), supermarkets, self-service stores (superettes + minimarkets), and discount stores. Scanner data has been widely used in the literature to investigate the demand for food products [14,38,39,40,41,42,43,44] and wine [27,45]. The advantage of using this type of revealed preference data is that it refers to consumers’ actual purchases, resulting in a more reliable and useful analysis of market trends [40,41].

Store scanner data are collected at cash registers and identify the products, quantities sold, and prices paid. Each product sold is defined by an EAN code. These data thus supply the sum of the monthly sales in terms of value, volume, and unit, both at discounted and base prices. Furthermore, for each EAN code, the database reports additional information, such as year and month of sales, denomination (if available), brand, winegrower, colour (red, white, or rosé), geographical indication, type of format (e.g., plastic, bag-in-box, carton), volume of format (e.g., 0.75 liters, 1 liter, 1.5 liters), and grape variety.

The collected data were extracted for the wine sales recorded in March–April (termed b1) and June–July (termed b2) of 2020, the year of the pandemic’s appearance, and the total sales of the same periods of the single years from 2015 to 2019. These selected data were deeply analyzed to observe annual variations, following the methodology applied in the literature for exploring consumption trends during the COVID-19 pandemic. In fact, several scholars have analyzed purchasing patterns using secondary data (such as IRI data), exploring variations with respect to the previous year and consequently drawing inferences. Arellana et al. [46] studied the impacts on transport systems (focusing on air, freight, and urban transport demand) due to the COVID-19 outbreak in Colombia. Coluccia et al. [16] offered an analysis on the status of the agri-food sector during the pandemic, with an exploration of consumers’ demands by analyzing retail sales value and the consumer food price index. Cavallo et al. [15] presented an overview of the main changes in food habits during the lockdown, analyzing purchases of different food products compared to 2019. Using food purchase data, Del Pozo de la Calle et al. [17] described the nutritional quality of diets in Spanish households during the first COVID-19 epidemic wave and detected variations with respect to the same period of 2019. Bracale and Vaccaro [14] analyzed IRI data of 30 categories of food products during the first period of the pandemic (from 23 February to 29 March 2020) and explored the differences with sales from the same period of 2019. This established methodology represents a useful tool for implementing early analyses and evidencing changing trends on purchasing food habits caused by this unprecedented pandemic that the world is currently experiencing. At the same time, these first contributions have paved the way for further research on the food market. In contrast with the previously mentioned literature, instead of considering only data from 2019 (the year immediately preceding the pandemic), we have expanded our analyses to 2015, in order to draw precise conclusions on purchasing trends and to underline the presence of trends. Specifically, we compared the sales of 2020 b1, in the lockdown period, with the sales of b1 of the respective previous years. To study the situation of the post-lockdown period, a twofold comparison was made: the first between the 2020 b2 data and the data of b1 of the same year; the second between the 2020 b2 data and the b2 data of the previous years. This twofold examination made it possible to verify whether, after lockdown, the wine sales in MMR maintained the trends verified during the lockdown, or whether they instead fell back in line with the trends of previous years.

## 4. Results

### 4.1. Wine Purchases during Lockdown

To explore the wine purchases during the lockdown period and answer research questions Q1–Q4, we examined the sales trends of wine from 2015 to 2020 in the March–April (b1) period.

**Figure 2:**
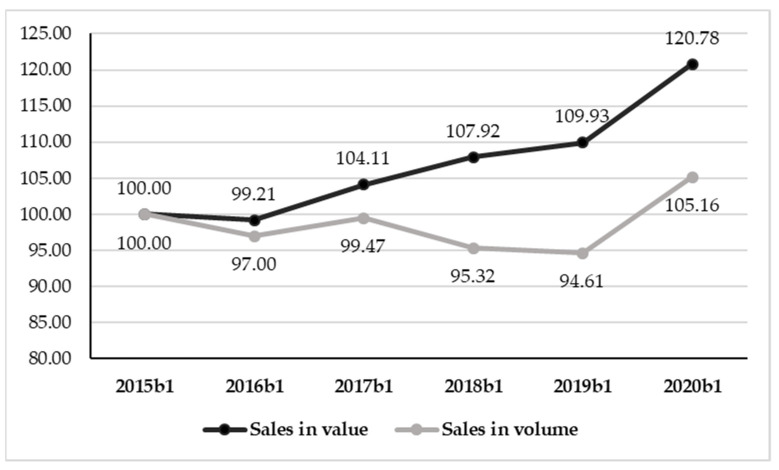
Indexes of wine sales in value and volume in MMR from 2015 to 2020 in b1 (base 100 = 2015b1).

Considering Q1, the total sales in the 2020 b1 period showed an increase in sales in terms of both value and volume, with different performances compared to the previous years (Figure 2).

In fact, even though from 2016 the sales in terms of value showed a constant increase, 3.5% annually on average, in 2020 they nosed up to EUR 316.32 Million, thus recording +20.8% compared to 2015 b1 and +9.98% compared to 2019 b1 (more than double the food sales [47]). The performance of sales in terms of volume in 2020 was even more surprising. After a seesaw trend in 2015–2017, the sold volumes diminished, in 2019 reaching 83.98 million liters. The lockdown reversed this trend, with an increase in sales in terms of volume of 11.2% over the previous year, and reaching a total of 93.35 million liters. In particular, the rate of increase for the sales in volume in 2020 b1 was similar to that of the sales in value.

To answer Q2, we analyzed the trends in wine sales by price range (Table 2).

The greatest percentage increase in terms of sales in value occurred for the wines priced between 6 and 14 EUR/liter (+14.3% compared to 2019 b1). However, in 2020, the *basic wines* (0–3 EUR/liter) seemed to show the most surprising result. Although in fact they represent the second largest percentage increase in absolute value (+12.5% compared to 2019 b1), this increase is also accompanied by a reversal of trends compared to the sales of the previous years. The sales in value of *basic wine* were in fact constantly declining, turning around only in 2020 until they reached EUR 99,600,000. The data that emerged in this analysis showed interesting trends also for products that fell within other price ranges. Wines priced between 3–14 EUR/liter confirmed the positive trend of the previous years, though with different increase rates. Although the 3–6 EUR/liter range, following the stagnation of 2019 (+0.7% compared to 2018 b1) returned to a growth in the percentage value similar to that of previous years (+7.1% compared to 2019 b1), the 6–14 EUR/liter range in 2020 showed the greatest growth rate compared to previous years. A change in the trend occurred, however, for the wines priced over 14 EUR/liter which, after positive variations though with different magnitudes, recorded a reduction in sales in 2020 b1. In particular, the wines priced between EUR 14 and EUR 25 per liter scored −7.7% compared to 2019 b1, whereas those priced over EUR 25 decreased by 19.0%.

Regarding Q3, we explored the sales of various formats of wine in the 0–3 EUR/liter range and their evolution from 2015 (Table 3).

Although it was the most sold format in this range, the carton was the format that exhibited the lowest rate of increase in sales. In fact, after a strong decline in 2016, followed by a seesaw trend in the years following, in 2020 b1 it recorded an increase of +10.5%, reaching EUR 39.76 million. However, the major increases in terms of sales in value were recorded for the large formats, that is, those of two liters or more. In this sense, in 2020 b1 the bag-in-box format showed surprising results. In fact, even though in previous years the trend had always been positive, despite different annual variations, indicating the consumer’s greater appreciation of this product, 2020 b1 recorded an increase of 52% compared to 2019 b1 with EUR 6.97 million in sales. Wine in plastic containers, sold especially in the three- and five-liter formats, marked an increase of +34.9% over 2019 b1. Wine in glass containers with a higher capacity than 0.75 liters, that is, the two types of “glass from 0.76 to 2 liters” and “glass more than 2 liters” (type mainly formed by five-liter demijohns), in 2020 b1 recorded a net sales increase (+22.0% and +38.1% respectively compared to 2019 b1), reversing the declining trend of the previous years. The category “glass up to 0.75 liters” (where 0.75 liter bottles constitute 99% of the total sales of this format) is the only one that recorded a reduction in 2020 b1 (−4.9% compared to 2019 b1). Even though this category showed an increment in 2019 b1, the reduction in 2020 b1 followed the general decrement that had characterized this format on the market since 2015. This result draws particular interest in a sales framework, where all the formats exhibited positive performances.

To explore the 0.75 liter glass bottle format and answer Q4, we first analyzed the trends of total wine purchases in 0.75 liter glass bottles and by price range (Table 4).

The value of the total sales of this format grew in 2020, rising to EUR 234.11 million. However, the growth rate with respect to the previous year (+6.8%) was similar to that of the entire wine sector. It is interesting to note, however, the different trends depending on the price range. The bottles belonging to the *ultra-premium category* (with prices higher than 14 EUR/liter) underwent a reduction of sales in value. This reduction was unexpected: after a constant growth in the previous period, in 2020 the bottles priced between 14 and 25 EUR/liter took up position at −7.4%, and those of the *great wines* (>25 EUR/liter), at −19.6% with respect to 2019. Table 4 also provides interesting results concerning the intermediate ranges of 3–6 and 6–14 EUR/liter. The sales confirm the positive trend assumed in the previous years and show a steady increase in the two months of lockdown compared to 2019, respectively of +6.1% and +14.8%.

Concerning the promotional sales in value of the 0.75 liter bottles (Table 5), in 2020 b1 the data showed a 17% reduction of sales in promotion compared to the previous year.

**Figure 3:**
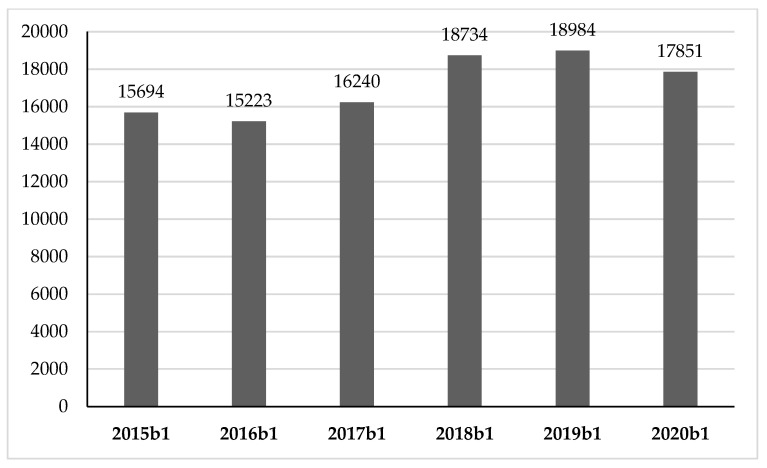
Number of EAN codes of 0.75 liter bottles sold.

For exploring whether purchases of 0.75 liter bottles of wine increased in terms of EAN codes, we conducted an analysis, of which the results are presented in Figure 3. As of 2016, we witnessed an increase in the number of different labels purchased by consumers, but the year 2020 recorded a halt, with a 6.0% decrease over the previous year.

The trend of average prices for bottled sales was also analyzed (Table 6). The price trends since 2015 have always exhibited positive signs, settling on 4.91 EUR/liter in 2019 (+2.2% with respect to 2018) and 4.99 EUR/liter in 2020. This increase (+1.7%) during lockdown seems to follow the trend of the previous years.

### 4.2. Wine Purchases in the Post-Lockdown Period

**Figure 4:**
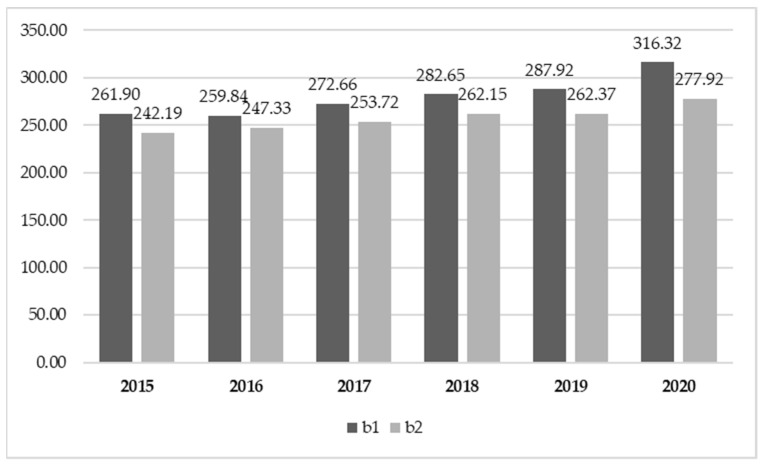
Wine sales trend in value on the Italian MMR (millions of EUR).

The final research questions (Q5) explored the possible changes in purchasing trends in June–July 2020 (b2) compared to the lockdown period (2020 b1) and the same period of previous years. The first significant result we wish to stress concerns the sales in value (Figure 4).

In 2020 b2, the post-lockdown two-month period, we noted a more marked increase in wine sales compared to previous years. Although in fact, as of 2016 b2, we had recorded average annual increases of 2.0% compared to the b2 of the previous year, in the post-lockdown 2-month period, sales showed an increase of +5.9%. This result seems particularly significant, despite sales having suffered the usual decrease compared to the sales of b1, owing to the season. In fact, as pointed out by Contini et al. [18], with the arrival of summer, wine sales recorded a decline compared to the first four-month period of the same year. These performances indicate that, in spite of the lifting of restrictions, the Italians maintained high wine purchases in MMR, though attenuated by the arrival of summer.

**Figure 5:**
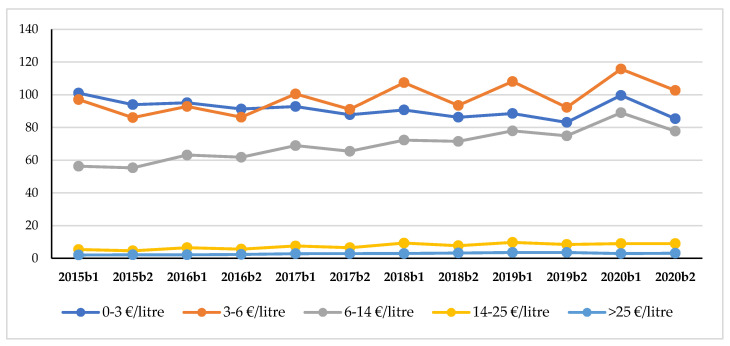
Wine sales in value (millions of EUR) by price range.

Analyzing trends by price range (Figure 5 and Table 7), it emerges that in the post-lockdown period, the sales in the value of wines priced up to 3 EUR/liter increased over the previous year (+2.6%), despite the seasonal decline compared to b1, after years of a negative trend (−3% on the average).

This result underlines that the purchases of *basic wine,* although decreased compared the lockdown, grew +2.6% with respect to 2019 b2. Taking a closer look at the trends between sales by price range, we must also stress the performance of the 3–6 EUR/liter category, which is to say the category that as of 2017 comprised most of the sales in value. Following the decrease in sales recorded in 2019 b2 (−1.3%) compared to b2 of the previous year, the sales of this price range changed direction during the post-lockdown period, also recording a higher percentage increase (+11.2%).

**Figure 6:**
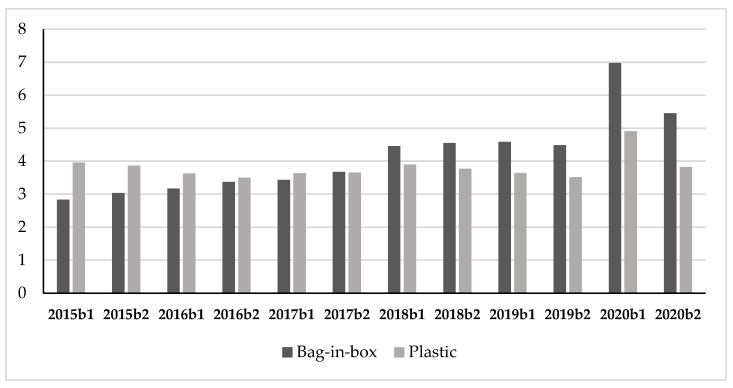
Wine sales in value (million EUR) of bag-in-box and plastic in the 0–3 EUR/liter price range.

Figure 6 and Table 8 shows the values and the trends of the bag-in-box and plastic formats in the 0–3 EUR/liter range in the post-lockdown period. Despite the marked decrease in sales compared to 2020 b1, owing to the seasonality and the fact that during lockdown they had seen a strong increase of sales compared to the past, these formats recorded marked sales increases compared to 2019 b2, respectively of +21.7% for the bag-in-box, and + 8.8% for the plastic container. Therefore, even after lockdown, consumers maintained the habit of purchasing wine in the *basic* price range in the bag-in-box and plastic formats.

The analysis of the sales of wine in 0.75 liter glass bottles shows that the average prices per liter did not present significant variations (+0.5%) in 2020 b2 compared to 2019 b2.

The 2020 b2 results indicate for *great wines* (Table 9) a substantial drop in sales value (−13.6% compared to 2019 b2). This fact contrasts with the past trends, in which, in the 2016–2019 b2 period, we always witnessed an increase in sales compared to the previous years (13.9% on average). In fact, in the summer months, consumers are used to purchasing more expensive wines. The results demonstrated that in 2020 b2 consumers opted for lower ranges, of which the trends were positive (for example, wines 3–6 EUR/liter, +11.3% compared to 2019 b2). We note, however, an increase in sales for *great wines* compared to lockdown, which is coherent with the seasonal positive trend for this price range.

Table 10 shows the trends of promotional sales of 0.75 liter bottles. Compared to 2019 b2, 2020 b2 recorded an increase in sales under promotion (+10.8%), confirming the positive trend encountered in the past. It is interesting to note, however, the increase with respect to the lockdown period. The usual decline in promotional sales of the summer season with respect to the spring months, which had always occurred in previous years, was reversed in 2020 with an increase of +7.7%.

Therefore, several purchasing trends encountered in 2020 b2 changed compared to the b2 period in the previous years. This is shown, in particular, by the increase in overall wine sales, the increase in wines priced below 3 EUR/liter and bottles priced between 3 and 6 EUR/liter, the boom of wine sales in the bag-in-box and plastic formats, and the drop in sales of bottles priced above 25 EUR/liter. These trends examined in the post-lockdown followed the purchasing habits acquired by consumers during the lockdown.

## 5. Discussions

This study provided evidence on different consumers’ purchasing patterns for wine compared to the previous years. Similar to other foodstuffs [15], the dynamics set off by the pandemic have modified the value and type of wine purchases. Even if wine has lost that essential role in the Italian meal that it played in the past, it still remains an important component of Italian food expenditure [48]. During the lockdown, wine purchased on the MMR channel increased by 11.2% in volume and 9.9% in value, more than double the value of the total food sales observed by Nielsen [47]. The closing of bars and restaurants could have had a role in the increasing performance of wine sales on the MMR channel, as could the increased number of people working remotely from home during lockdown [7]. In fact, remote workers have shown the highest increase in the consumption frequency of wine (+17.9%) [49]. This eating pattern established is also related to the increase in the purchase of comfort foods, types of food that give psychological comfort in periods of stressful events and are therefore purchased under an emotional impulse, rather than as a real necessity [15]. However, our results may be in part attenuated by the increment in wine sales from different channels, i.e., small grocery stores and e-commerce, as evidenced by IRI [11].

We witnessed an increase in sales for wines priced below 3 EUR/liter (+12.5%), a boom of wine sales in the bag-in-box and plastic formats (respectively, +52.0% and +34.9%), and a reduction in the sales of bottles belonging to the *ultra-premium category* (priced over 14 EUR/liter). The increase in sales of low-priced wines such as the reduction of wine priced over 14 EUR/liter may find an explanation in the economic difficulties that emerged with the pandemic. The economic shocks due to COVID-19 and the sharp increase in unemployment have probably led some consumers to place more importance on price in food sales compared to other factors [50]. Wine, not being an essential good, is characterized by a demand that is more sensitive to socio-economic dynamics [18], and the price results in being the most important attribute in the purchasing act during an economic crisis [51]. Together with the financial crisis, the government has imposed some restrictions during the lockdown by forbidding celebrations or meetings with relatives. Since for special occasions consumers are willing to pay higher prices for a bottle of wine [52,53], these social limitations contribute to the decline in wine sales in the *super-premium* and *great* categories, as also emerged in a study by Vergamini et al. [54]. The negative trend of *great wines* persisted even in the period after lockdown, with a lower sales value compared to the levels of the previous years.

Differently from the general trends analyzed for wine, the 0.75 liter glass bottles priced under 3 EUR/liter record a reduction in their sales during the lockdown. Albeit with the necessary caution, on the basis of the results, the decline of bottled wine sales in the under 3 EUR/liter price range could be due to the fact that, during the lockdown, when oriented towards a *basic wine*, the consumer preferred other formats such as carton or bag-in-box. In fact, further analyses on the sales volume data confirmed this trend in sales value. Although wine bottles showed a decrease in sales in volume equal to −6.0%, bag-in-box and plastic exhibited a growth, respectively, of +42.6% and +32.2%. The explosion of sales in MMR of large formats such as bag-in-box and plastic is one of the most surprising trends of consumption acquired during the two-month periods. The forced change in the consumers’ daily eating habits during the lockdown, characterized by an increase in the number of meals eaten at home, may have promoted the growth of the purchases of “daily consumption wine”, as these formats are traditionally considered [55]. In addition, in other countries researchers have observed an orientation towards large formats during the pandemic, namely, consumers bought larger-sized alcoholic beverages in consequence of the less frequent shopping trips due to concern of contagion and restrictions [29]. The literature seems to confirm that, at the supermarket, the consumer opted for easily transportable wine containers that, at the same time, ensured good product preservability. Although in the past, particularly the bag-in-box format was the target of prejudice and considered with contempt as a metallized plastic bag in a cardboard box, the lockdown has caused its sales to skyrocket thanks to its capability to respond to consumers’ temporary demands in terms of practicality, convenience, and the need to stock up on the product. Not only is this format a good buy for money, but it also makes it possible to preserve the wine well for days after opening, thanks to the absence of light and contact with the air [56]. Moreover, bag-in-box packaging is often related to the concept of sustainability, biodegradability, and the use of recyclable materials. These qualities, which Italian consumers are increasingly more interested in [57,58,59], could further boost their loyalty and attract new customers. The appreciation for bag-in-box formats is also confirmed by the sales of the post-lockdown period. If this format did not find general support among consumers before the pandemic, their purchases due to the contingent situation and their satisfaction with the taste could have modified the consumers’ opinion on the bag-in-box format, convincing them to buy it again.

Despite reopenings in the restaurant sector, in the post-lockdown period the value of wine sales on the MMR channel, as well as the market share of low-priced wines and large formats, was higher compared to the same period in previous years, confirming the sales trend evidenced in the lockdown.

The negative trend of promotional sales in value of the 0.75 liter bottles (−17%) is worthy of particular attention. We can trace this outcome back to the general negative trend of all food promotional sales in the Italian MMR [36]. In an investigation into the possible causes of this phenomenon conducted during the lockdown, different reasons were given by the directors of supermarket chains [60]. According to some of these directors, the decrease in food promotion purchases were due to the change in the purchasing habits of consumers who shopped quickly, paying more attention to safety than promotions and focusing on essential products that were not subject to promotions. Instead, another group of supermarkets directors has stated that promotions were modulated according to the availability of the industry that could not cope with the required volumes, because in March food purchases increased by a quarter [60]. If in MMR during the lockdown the value of the purchases of wine bottles under promotion decreased, the following period did not confirm this trend. In fact, in the b2 2020 period, promotional wine sales increased, contradicting the usual seasonal decline for the months of June and July traditionally observed in the previous years and evidenced by the literature.

## 6. Conclusions

Our study provides a detailed analysis of wine sales during the COVID-19 pandemic on the MMR channel throughout Italy. Since MMR traditionally represents the main distribution channel for wine and the HoReCa sector was closed due to the restrictive measures during the lockdown, this study provides insights on a very relevant part of the wine market in this pandemic period. In particular, the analysis of the sales in the period of the lockdown (March–April 2020) and post-lockdown (June–July 2020) enabled us to outline a detailed and effective evolution framework of this sector, compared to the previous years. The scenario depicted shows the effects on wine sales of a global crisis that is unprecedented and not fully comparable with previous economic or health crises. To our knowledge, no previous studies have so thoroughly explored the total wine sales of this market channel during this unique period. We believe that such an analysis of the dynamics of wine purchases made during the pandemic could shape our expectations for the evolution of the future wine market.

Even though the COVID-19 virus is increasingly under control, it has still not been overcome. The persisting outbreak and the restrictions that are frequently reintroduced (although less severe compared to the lockdown period) mean these new purchasing patterns acquired with the pandemic could be assimilated as new everyday habits. The transformed structure of wine demand provides marketers with some indications on the strategies for facing these new shopping paradigms. In the case of bag-in-box wines, producers are encouraged to organize testing sessions or offer free samples in supermarkets to try the product and acquire consumers’ loyalty. Considering the increasing consumer agreement with bag-in-box wine, winegrowers could resort to this type of packaging more often, characterizing the product with aspects aimed at supply chain traceability and environmental sustainability in the packaging. Moreover, producers of *premium* wines could think about expanding their supply and introduce the bag-in-box format on the market with a higher-priced wine, according to the regulations of denominations of origin. As far as retailers are concerned, the growing interest of consumers in this type of wine can be used as a potential lever to boost customer loyalty and attract new customers, also publicized by means of promotional campaigns. Alternative package formats and materials, such as bag-in-box packaging, are also particularly appreciated by millennials [61].

The framework on wine sales and the changing trends during the pandemic evidenced in this study represent an early contribution on the evolution of the wine market and can constitute a point of departure for further studies undertaking more thorough analyses using other methodologies. Our study focused on the first period of the pandemic, exploring the wine sales until July. It would be useful to expand the analysis by including the data for the same period of 2021, in which the mass vaccination campaign against COVID-19 allowed a gradual return to a normal way of life. These longer time series could allow a better comprehension of the evolution of consumers’ purchasing patterns.

Our outcomes suggest to researchers the need to implement studies with testing sessions or free samples to elicit consumers’ preferences and willingness to pay, especially for products with alternative packaging, such as bag-in-box wines, or other types of food which meet with widespread scepticism. In fact, the evidence that emerged from hypothetical studies could be reversed by experimental studies involving a real experience with the product and that allow respondents to value the characteristics of the food considered in the study. The methodology used in this study could also provide the starting point for a more complete analysis of the food market. Indeed, the changes in sales that emerged for wine during the pandemic may have affected other food products as well. An example is beer, which is increasingly appreciated by consumers and can be subject to a substitution effect with wine. These analyses should be conducted considering the temporal evolution of the pandemic to provide better evidence on the emerging trends. To complete the picture of the wine market during the pandemic, future studies should integrate sales data considering segmentation variables such as economic conditions, working habits during the lockdown, and psychographic characteristics.

Our exploratory study has some limitations that should be recognized. The main limitation of this study was the use of a secondary data source, which was context-specific and not generalizable to other countries, characterized by different restrictions imposed and consumer characteristics. In fact, given the different spread of the pandemic in the various countries over time, each government has adopted restrictive measures and limitations according to its own sanitary situation. In Italy, which was the first Western country to establish the lockdown, the restrictive measures may have lasted longer or been stricter, affecting purchasing habits in a different way than in other countries. Concerning our data, the database used did not include the data on the sales related to sparkling wines. This therefore prevented us from further investigating this type of wine, which represents a part of wine sales at supermarkets, and which is traditionally purchased for special occasions such as meetings with friends and relatives. Among the limits of this study, we wish to include the impossibility of conducting analyses of socio-economic categories of consumers. Although store scanner data, referring to sales aggregated on the territory by product, make it possible to survey general trends, they do not allow us to conduct analyses on the socio-economic characteristics of purchasers. For this reason, in this study it was not possible to exploit the traits of the buyers and the motivations that led to the choice of certain types of wine.

## Figures and Tables

**Table 1:** Overall picture of the Italian wine market in MMR from 2014 to 2020.

	2014	2015	2016	2017	2018	2019	2020
Sales in value (Millions of EUR)	1505.4	1540.3	1556.7	1603.1	1644.3	1669.0	1793.5
Sales in volume (Millions of liters)	505.1	511.3	506.0	508.7	483.7	485.7	509.1
Price (EUR/liter)	2.98	3.01	3.08	3.15	3.40	3.44	3.52
*Sales by format (Millions of EUR)*							
Glass up to 0.75 liters	1065.5	1116.5	1163.1	1216.6	1247.8	1289.0	1393.4
Other formats	173.2	170.3	150.5	143.7	138.3	127.7	133.9
Carton	227.5	214.4	202.2	199.9	210.1	204.2	207.5
Bag-in-box	15.9	17.2	19.2	21.0	25.2	26.9	35.3
Plastic	23.4	21.9	21.7	21.9	23.0	21.2	23.5

Source: Unione Italiana Vini [22] on IRI data.

**Table 2:** Sales in the value of wine by price ranges (Millions of EUR).

Price Ranges	2015 b1	2016 b1	2017 b1	2018 b1	2019 b1	2020 b1	Var. 2019 b1/2020 b1
0–3 EUR/liter	101.1	95.1	92.8	90.7	88.6	99.6	+12.5%
3–6 EUR/liter	97.1	92.9	100.5	107.4	108.1	115.7	+7.1%
6–14 EUR/liter	56.3	63.2	68.9	72.3	77.9	89.1	+14.3%
14–25 EUR/liter	5.4	6.5	7.6	9.3	9.7	9.0	−7.7%
>25 EUR/liter	2.1	2.2	2.8	2.9	3.6	2.9	−19.0%

**Table 3:** Sales in value of wine in the 0–3 EUR/liter range by format (millions of EUR).

Formats	2015 b1	2016 b1	2017 b1	2018 b1	2019 b1	2020 b1	Var. 2019 b1/2020 b1
Bag-in-box	2.84	3.17	3.43	4.46	4.59	6.97	+52.0%
Carton	37.92	35.22	35.45	36.50	35.97	39.76	+10.5%
Plastic	3.96	3.63	3.64	3.90	3.64	4.91	+34.9%
Glass more than 2 liters	7.01	5.80	5.56	5.18	4.84	6.68	+38.1%
Glass from 0.76 to 2 liters	19.05	17.09	16.01	15.52	13.83	16.87	+22.0%
Glass up to 0.75 liter	30.30	30.24	28.74	25.18	25.69	24.43	−4.9%

**Table 4:** Wine sales in value of 0.75 liter bottles by price range (millions of EUR).

Price Range	2015 b1	2016 b1	2017 b1	2018 b1	2019 b1	2020 b1	Var. 2019 b1/2020 b1
0–3 EUR/liter	30.30	30.24	28.74	25.18	25.69	24.43	−4.9%
3–6 EUR/liter	93.66	89.88	97.36	103.84	104.24	110.63	+6.1%
6–14 EUR/liter	55.08	61.60	67.27	70.67	76.19	87.46	+14.8%
14–25 EUR/liter	5.22	6.30	7.34	9.07	9.50	8.80	−7.4%
>25 EUR/liter	1.97	2.08	2.74	2.84	3.48	2.80	−19.6%
**Total**	186.23	190.10	203.45	211.59	219.10	234.11	+6.8%

**Table 5:** Sales in value in promotion of wine in 0.75 liter bottles (millions of EUR).

	2015 b1	2016 b1	2017 b1	2018 b1	2019 b1	2020 b1	Var. 2019 b1/2020 b1
**Total**	82.56	84.36	87.71	87.17	93.64	77.78	−17.0%

**Table 6:** Average price of wine in a 0.75 liter bottle (EUR/liter).

	2015 b1	2016 b1	2017 b1	2018 b1	2019 b1	2020 b1	Var. 2019 b1/2020 b1
Total	4.37	4.46	4.57	4.81	4.91	4.99	+1.7%

**Table 7:** Percentage change of wine sales in value by price range compared to the previous period: for each year, (*i*) the variation of b2 compared to b1, and (*ii*) the variation of b2 compared to b2 of the previous year.

Price Range		2015	2016	2017	2018	2019	2020
0–3 EUR/liter	*i*	−7.0%	−4.1%	−5.4%	−5.0%	−6.1%	−14.3%
	*ii*	-	−2.8%	−3.8%	−1.8%	−3.6%	+2.6%
3–6 EUR/liter	*i*	−11.3%	−7.1%	−9.4%	−13.0%	−14.6%	−11.3%
	*ii*	-	+0.2%	+5.6%	+2.6%	−1.3%	+11.2%
6–14 EUR/liter	*i*	−1.7%	−2.2%	−5.0%	−1.1%	−3.9%	−12.7%
	*ii*	-	+11.6%	+6.0%	+9.2%	+4.7%	+3.9%
14–25 EUR/liter	*i*	−13.4%	−13.4%	−14.1%	−17.2%	−13.1%	+0.2%
	*ii*	-	+21.0%	+15.4%	+18.8%	+9.9%	+6.5%
>25 EUR/liter	*i*	+4.8%	+8.7%	+1.1%	+10.4%	+0.0%	+7.7%
	*ii*	-	+9.6%	+21.3%	+13.0%	+10.4%	−12.8%

**Table 8:** Percentage change of wine sales in value by format in the 0–3 EUR/liter price range compared to the previous period: for each year, (*i*) the variation of b2 compared to b1, and (*ii*) the variation of b2 compared to b2 of the previous year.

Format		2015	2016	2017	2018	2019	2020
Bag-in-box	*i*	+7.2%	+6.2%	+7.2%	+2.2%	−2.3%	−21.8%
	*ii*	-	+11.0%	+9.1%	+23.7%	−1.5%	+21.7%
Plastic	*i*	−2.3%	−3.5%	+0.6%	−3.4%	−3.5%	−22.2%
	*ii*	-	−9.4%	+4.5%	+3.0%	−6.7%	+8.8%

**Table 9:** Percentage change of wine sales in value in 0.75 liter bottles by price range compared to the previous period: for each year, (*i*) the variation of b2 compared to b1, and (*ii*) the variation of b2 compared to b2 of the previous year.

Price Range		2015	2016	2017	2018	2019	2020
0–3 EUR/liter	*i*	−8.8%	−4.3%	−6.4%	−6.0%	−4.9%	+1.5%
	*ii*	-	+4.7%	−7.0%	−12.0%	+3.2%	+1.4%
3–6 EUR/liter	*i*	−11.5%	−7.3%	−9.5%	−13.2%	−14.6%	−10.4%
	*ii*	-	+0.5%	+5.8%	+2.3%	−1.3%	+11.3%
6–14 EUR/liter	*i*	−1.7%	-2.0%	−5.0%	−1.1%	−3.9%	−12.6%
	*ii*	-	+11.4%	+6.0%	+9.3%	+4.7%	+4.4%
14–25 EUR/liter	*i*	−13.1%	−13.1%	−13.7%	−17.2%	−12.9%	+0.4%
	*ii*	-	+20.6%	+15.7%	+18.5%	+10.3%	+6.8%
>25 EUR/liter	*i*	+6.0%	+9.5%	+1.8%	+11.5%	+0.8%	+8.3%
	*ii*	-	+9.2%	+22.1%	+13.4%	+10.9%	−13.6%

**Table 10:** Percentage change in wine sales in value in 0.75 liter bottles under promotion compared to the previous period: for each year, (*i*) the variation of b2 compared to b1, and (*ii*) the variation of b2 compared to b2 of the previous year.

		2015	2016	2017	2018	2019	2020
Bottle 0.75 liter	*i*	−16.7%	−17.5%	−16.8%	−17.0%	−19.2%	+7.7%
	*ii*	-	+1.2%	+4.8%	-0.9%	+4.6%	+10.8%

## Data Availability

Not applicable.
